# A Case of Hypopharyngeal Cancer Associated With Fanconi Anemia: A Helical Tomotherapy Experience

**DOI:** 10.7759/cureus.19386

**Published:** 2021-11-09

**Authors:** Gizem Kavak, Semih Basci, Esra Türker Kekilli, Mehmet S Dal, Ebru Karakaya

**Affiliations:** 1 Radiation Oncology, University of Health Sciences, Ankara Oncology Training and Research Hospital, Ankara, TUR; 2 Hematology, University of Health Sciences, Ankara Oncology Training and Research Hospital, Ankara, TUR

**Keywords:** fanconi anemia, helical imrt, tomotherapy, cetuximab, head and neck squamous cell cancer, radiation therapy, aplastic anemia, concomitant chemo-radiation therapy

## Abstract

Fanconi anemia (FA) is a disease that could be accompanied by multiple abnormalities, such as growth retardation, bone marrow abnormalities, and cancer susceptibility. Among the FA patients, head and neck squamous cell cancer (HNSCC) is the most observed solid cancer. The life expectancy of patients with FA has increased with recent medical advances. Furthermore, HNSCC is diagnosed in 3% of FA patients, and half of these patients die because of their HNSCC. The median age of HNSCC patients with FA is 31, and according to the literature HNSCC incidence of FA, patients is more than 700-fold of the normal population. Here, we reported the treatment details and challenges we faced during hypopharyngeal cancer treatment in a FA patient.

## Introduction

Fanconi anemia (FA) is a genetic bone marrow failure syndrome characterized by pancytopenia. It is prone to malignancy and physical abnormalities, including short stature, microcephaly, developmental delay, café-au-lait skin lesions, and malformations. Overall, an average of one out of 136000 newborns has Fanconi anemia, and it varies from one in 100000 to 250000 births [1]. Diagnosis is usually done in childhood, but sometimes diagnostic delays and variable disease manifestations are common, and some individuals may be diagnosed with FA in adulthood [2]. The average survival of individuals with FA was 21 years old before the 21^st^ century; afterward, remarkable improvements like stem cell transplantation, gene therapy, epigenetic targeting occurred in the survival of these patients [3,4]. Allogeneic hematopoietic cell transplantation (HCT) is the preferred treatment option in children with idiopathic severe aplastic anemia and FA when feasible [5]. 

FA is caused by a genetic defect in a cluster of proteins capable of DNA repair through homologous recombination [6]. Because of the genetic defect in DNA repair, FA patients are very prone to side effects of radiotherapy (RT) and cancer-treating drugs by DNA cross-linking, like mitomycin C [7]. Patients with FA are at a high risk of developing a malignancy comprising HNSCC, myelodysplastic syndrome, and acute myelocytic leukemia [8-10]. Head and neck cancers in these patients have an earlier onset without other risk factors (such as tobacco, alcohol). The management of patients with head and neck cancer caused by FA is challenging due to increased susceptibility to radiation therapy complications [7]. The reason why we want to present this case is that the treatment of patients with FA and HNSCC is challenging and rare, based on case series in the literature [7,10,11].

## Case presentation

A 25-year-old woman was admitted due to complaining of difficulty in swallowing. She was the youngest of the five siblings, also a non-smoker and non-drinker. She was a child of a consanguineous marriage as her father and mother are cousins. Furthermore, when she was five years old, she was diagnosed with Fanconi anemia. To confirm the diagnosis of FA, we communicated with the related university hospital for the records of the patients 20 years ago. They approved the diagnosis of FA with some peripheric blood sample studies without giving details. They had offered bone marrow transplantation, which the parents had not approved of. She had no major symptoms for 20 years, and the disease was under control. But the patient did not go to regular hospital check-ups. The patient's first notable characteristic was growth retardation manifested by short stature, microcephaly, and microphthalmia. Afterward, she had swallowing difficulty for the last two years and had lost around 10 kg in the last six months.

**Figure 1:**
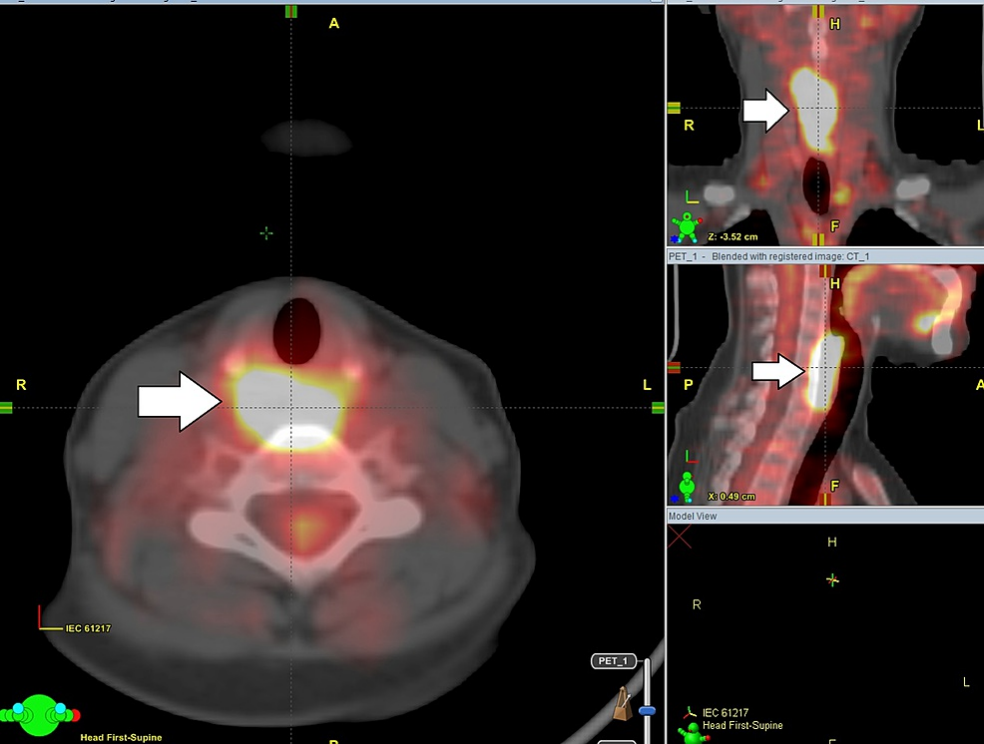
Positron emission tomography imaging The lesion indicated by the arrows is in the hypopharynx, starting from the right side of the oropharynx and extending to the proximal esophagus (SUV max: 8.5).

The patient was referred to the gastroenterology department due to swallowing problems. In the endoscopic examination, stenosis was observed in the hypopharynx that restricts the passage of the scope follows through. Positron emission tomography (PET-CT) scan showed multiple lymphadenopathies in the bilateral deep cervical lymph nodes (standardized uptake value [SUV] max: 8.8), and prominent pathological 18F-Florodeoksiglukoz (FDG) involvement beginning from the right-side oropharynx to the proximal esophagus (SUV max: 8.5) (Figure 1). The patient underwent endoscopy, tumoral formation in the hypopharynx leading to only 3 mm passage opening was detected. As a result of punch biopsy and pathological examination, squamous cell carcinoma of the hypopharynx was revealed.

**Figure 2:**
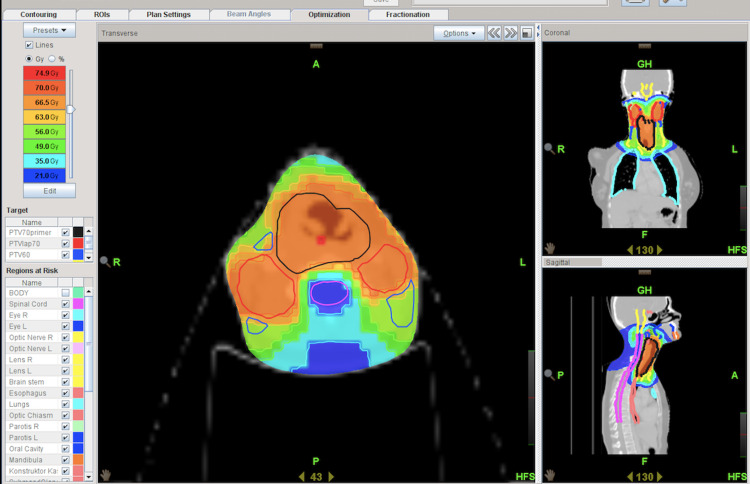
Helical Tomotherapy plan of the patient Blackline: planning target volume (PTV) 70 primary. Redline: PTV 70 lymphadenopathy. Dark blue line: PTV 60

The patient was diagnosed with T3N2cM0 hypopharyngeal cancer, and due to locally advanced disease, surgery was not considered; finally, she opted for curative radiotherapy (RT) with concomitant cetuximab chemotherapy. The RT of the patient was planned on a helical Tomotherapy system (Figure 2), including primary tumour and bilateral neck lymph nodes. At the beginning of RT, the complete blood count was near-normal, with only mild leukopenia (white blood count is 3,8x10^3cells/uL). Cetuximab loading dose could not be administered due to urgent treatment needs. Consequently, the first cetuximab dose 350 mg/m2 with RT was initiated. The remaining maintenance dosage was scheduled as 250 mg/m2 but could not be administered due to a sudden drop in blood count (white blood count to 0.48x10³cells/uL) after four days of initial cetuximab and 10 days of RT. Her treatment was terminated due to progressive cytopenia (Table 1), after receiving 21.2 Gy in 10 days for primary lesion. A peripheral blood smear was investigated, and it was concordant with severe pancytopenia as no atypical cells with rare leukocytes and platelets. To secure hematological recovery, G-CSF was initiated, erythrocyte and platelet transfusion was administered when necessary. Despite the daily G-CSF utilization, there was no significant change in blood counts. Moreover, the patient's dysphagia was worsened and a nasogastric tube was placed for feeding, in addition to parenteral nutritional support. Despite receiving nasal oxygen, the patient's condition worsened, on the 30^th^ day of the start of radiotherapy, and her oxygen saturation regressed, and she was intubated. The patient died 40 days after radiotherapy begin due to respiratory failure and sepsis.

**Table 1:** Common blood counts according to radiotherapy days Abnormal findings are shown in bold. D: days of radiotherapy. RT: radiotherapy. WBC: white blood count (normal value range: 4-10x10^3cells/uL). NEU: neutrophil (normal value range: 2-7x10^3cells/uL). HGB: hemoglobin (normal value range: 11-16 g/dL). PLT: platelet (normal value range: 100-300x10^3cells/uL). * First leukopenia observed, corresponding to the fourth day of cetuximab. ** With transfusion support

DAYS (RT)	WBC (x10^3cells/uL)	NEU (x10^3cells/uL)	HGB (g/dL)	PLT (x10^3cells/uL)
Basal blood count (day 0)	3,8	2,69	11,63	191
D6	3,4	2,09	11,47	178
D8	3,93	2,98	11,1	187
D10*	0,48	0,23	10,0	110
D11	0,05	0	10.2	82
D14	0,03	0	7,7	21
D18	0,06	0	6.5	2
D31	0,06	0	10,1**	16

## Discussion

According to IFAR (International FA Registry), the mean survival of FA patients was reported as less than 25 years in 2003. Also, 3% of 754 FA patients were identified to have an HNSCC [3]. So, FA patients are strongly prone to HNSCC development; moreover, they have an earlier onset for HNSCC [2]. According to the age distribution, head and neck scanning should be started before the age of 15, and a detailed examination should be performed periodically [5,12,13]. In agreement with the literature, our patient was 25 years old with no alcohol and smoking use when diagnosed with hypopharynx cancer.

Cancer management in patients with Fanconi anemia is a challenging process. Evidence regarding the subject is limited and available only in the context of case reports.

The genes giving way to the development of FA have a role in the FA DNA interstrand crosslink repair pathway, which functions to repair DNA crosslinks using elements of nucleotide excision, homologous recombination, and translation synthesis repair pathways [14]. The propensity of FA patients to develop cancer is well known. Until the 40 years old, 28% of FA patients expect to develop solid tumours [3]. According to Kaplan [2], two main defects are involved in developing malignancies consisting of head and neck squamous cell cancer in patients with FA: immunodeficiency and defective chromosomal stability. Increased spontaneous instability, for example, breaks, fragments, radials, and dicentric chromosomes, are also identified in these patients [2]. Therefore, FA patients show an impaired capacity to repair DNA crosslinks. However, FA patients may need to receive chemotherapy and radiotherapy. The first example is hematopoietic stem cell transplantation preparation regimens used in the management of aplastic anemia caused by FA. These preparative regimens can cause significant toxicity in FA patients. It is known that FA patients are hypersensitive to genotoxic agents such as busulfan, cyclophosphamide, and ionizing radiation. To avoid this toxicity, the focus should be on reducing the doses required for transplant preparation regimens, selecting non-genotoxic regimens, and using alternative conditioning regimens [5]. The second example of situations in which these patients will need to receive chemotherapy and radiotherapy is the development of cancer, as in our patient. Our patient was also considered surgically inoperable, and the remaining treatment option was concurrent chemoradiotherapy or radiotherapy alone.

The increased susceptibility due to defective DNA repair mechanism in HNSCC patients with FA may pose a problem in administering therapeutic doses of chemotherapy and radiotherapy. In a study by Gluckman et al., five patients diagnosed with Fanconi anemia underwent hematopoietic stem cell transplantation after a preparation regimen with cyclophosphamide, and severe cyclophosphamide-related toxicity was observed in all of them. The recommendation of the study was to avoid alkylating agents (such as cyclophosphamide, cisplatin) [15]. It is mentioned that cisplatin used in traditional chemoradiotherapy regimens may cause serious systemic complications in these patients, including irreversible aplastic anemia and catastrophic organ damage [2,16,17]. Therefore, concomitant cisplatin chemotherapy was not considered for our patient. In the study of Kutler et al., chemotherapy and radiotherapy are avoided if possible, and surgery is recommended for FA patients [2]. However, since our patient was accepted as inoperable by the surgeons, curative radiotherapy (RT) with concomitant cetuximab was decided for the patient. Cetuximab was added to strengthen the effectiveness of radiotherapy. Radiotherapy alone was not considered because the stage of the patient was T3N2cM0, and it was thought that only radiotherapy would be insufficient in terms of local and systemic control. And there are reports of concomitant treatment with cetuximab. In a case report published by Wong et al., the patient with FA who presented with squamous cell carcinoma of the tongue relapsed shortly after the first tumour was surgically treated. The stage of that patient who underwent previously total glossectomy and bilateral neck dissection was found to be T4aN2cM0 at the time of relapse. Considering the high relapse capacity, the patient was given cetuximab concurrently with radiotherapy. The patient received 6660 cGy radiotherapy to the high-risk region at the oral cavity and bilateral neck site; and 5040 to 6120 cGy to the low- and medium-risk bilateral neck region. The patient initially seemed to tolerate and respond well to the treatment, but within 10 weeks the disease relapsed and the patient died [10]. In a case series of nine patients by Beckham et al. [7], FA patients were operated on due to HNSCC, and four of them had received adjuvant RT with concurrent cetuximab, including one palliative treatment. Treatment had to be stopped early in one patient. Two out of three patients had disease recurrence, and one patient had a temporary response. These three patients had died seven, 12, and 26 months after diagnosis. As a result, the authors advise that in patients with adverse features, especially inoperable ones, adjuvant radiation with concurrent cetuximab may be feasible with careful monitoring from the very beginning, although local disease control is infrequent.[2].

The tolerability of RT is significantly impaired in patients with FA [2]. Hence, radiation should be used for high-risk cancer or in case of inoperability for head and neck malignancy. In the literature, Lustig et al. concluded that only five out of 17 FA patients from different case reports, treated for HNSCC by RT as primary or adjuvant treatment, tolerated RT well [11]. Besides, in a study by Kutler et al., 16 FA patients had undergone postoperative RT for HNSCC with conflicting results. Some patients faced complications at low doses of radiation; for example, side effects appeared in one patient even at 25 Gy. This patient had T4N0M0 buccal mucosa cancer; after 25 Gy postoperative RT, she developed sepsis and lost her life after two months of disease-free period. In the same study, a mean radiation dose of 50,5 Gy (lower than usual) was applied due to severe toxicity. Although these lower doses, disease-free survival in two and five years were 75% and 43%, so better than expected, suggesting that the tumour itself may also be more sensitive to radiation in patients with Fanconi anemia [2].

Our patient, unfortunately, could not tolerate RT with concurrent cetuximab. In fact, after adding cetuximab to radiotherapy hematologic values decreased dramatically. So, we have a suspicion about the reason for this decreasing if concomitant cetuximab and RT or cetuximab itself. Like the other case reports in the literature, the treatment of locally advanced HNSCC in FA patients is challenging. In our opinion, if RT has to be started due to inoperability, both RT and concomitant treatment doses could be decreased.

## Conclusions

When treating patients with Fanconi anemia and HNSCC, blood values may drop very quickly, especially during RT and concomitant therapy, even if the patient is in remission for FA-related aplastic anemia. Before and during the treatment, all decisions should be reviewed by the hematology department. The treatment decision should be taken in a multidisciplinary manner with the departments of otorhinolaryngology, medical oncology, radiation oncology, and hematology. When the head and neck cancer with the hematological disease is treated with RT and concurrent chemotherapy/targeted agents for a reason such as inoperability, blood values should be checked more frequently than normal before and during treatment. Before starting treatment, patients and their relatives should be well informed about possible adverse scenarios, especially hematological side effects. If possible, the dose of RT and concurrent medication should be tried to reduce. Data from the literature is limited to make any firm conclusions and that extensive studies are needed to validate the results from case series and reports.
